# Clinical and histology features as predictor of severity of mucormycosis in post-COVID-19 patients: An experience from a rural tertiary setting in Central India

**DOI:** 10.1177/20503121221074785

**Published:** 2022-02-03

**Authors:** Kavita Jain, Akshay Surana, Tej Singh Choudhary, Sudhakar Vaidya, Shirish Nandedkar, Manju Purohit

**Affiliations:** 1 Department of Pathology, R.D. Gardi Medical College, Ujjain, India; 2 Choudhary ENT Hospital, Ujjain, India; 3 Department of Ear Nose Throat, R.D. Gardi Medical College, Ujjain, India; 4 Department of Global Public Health Sciences, Karolinska Institutet, Stockholm, Sweden

**Keywords:** COVID-19, mucormycosis, histopathology

## Abstract

**Background:**

An upsurge in cases of rhinosinusitis with or without associated orbital and/or cerebral involvement by mucormycosis has been observed in post-COVID-19 patients. Our objective is to evaluate the clinical and histopathology features of these patients to determine the severity and develop a scoring on the extent on tissue invasion.

**Method:**

We prospectively enrolled and analyzed 95 post-COVID-19 patients who presented with the invasive mucormycosis of the head and neck region. Clinical and histology details were noted in predesigned forms. Various histology variables were graded from I to III to propose a scoring system for the severity of the disease.

**Results:**

Mucormycosis was common in males with a mean age of 46.8 ± 11 years. Facial pain was the most common presenting complaint and 77% of the patients were diabetic. Most cases (n = 59) showed a moderate degree of neutrophilic infiltrate with ⩾50% tissue necrosis and angioinvasion in three or more vessels with a fungal load of 2+/3+. Histology severity grade III was observed in patients who died from cerebral mucormycosis (n = 3) and septicemia (n = 2) and in patients who had undergone orbital exenteration (n = 6).

**Conclusion:**

The histopathology and severity score classification was directly correlated with the outcome of the patients. Further evaluation and a larger study will help to validate the proposed scoring for its clinical use in all forms and causes of mucormycosis.

## Introduction

Mucormycosis is a rapidly progressive potentially life-threatening opportunistic infection characterized by angioinvasion and infarction. It represents the third most common angioinvasive fungal infection after candidiasis and aspergillosis.^1^ Being angioinvasive, it spreads rapidly and may clinically present as rhino-orbito-cerebral mucormycosis (ROCM), pulmonary, disseminated, cutaneous, or gastrointestinal disease.^2^ Mucormycosis was increased in frequency in recent years, with a global annual incidence of 0.005–1.7 cases per million population per year and approximately 0.14 cases per 1000 population in India.^3,4^ India has been shown to be the most affected country by mucormycosis even before COVID-19 era^5^ and during the second wave of the COVID-19 pandemic the prevalence of mucormycosis was nearly 70 times higher than the global data^4,6^ and the Indian Health Ministry has advised all States to declare mucormycosis itself an epidemic.^7^

There is a complex interplay between type 2 diabetes mellitus, immunosuppressive therapy, and systemic immune alteration in COVID-19 infection that could have led to its increase in post-COVID-19 patients.^8^ The cclinical symptoms and signs of various forms of mucormycosis are also varied. The ROCM is the most severe invasive form of mucormycosis and its prognosis is generally poor with high morbidity and overall mortality of approximately 54%.^9^ The high morbidity and mortality of ROCM are linked to the delay in the diagnosis of this terrible invasive disease.^1^ Studies suggest that an early diagnosis, immediate intense antifungal therapy with amphotericin B, although known to be a highly nephrotoxic drug, an elaborate radical surgery for debridement of a local tissue that often leaves extreme physical disfigurement, and correction of the underlying factor are associated with favorable outcomes even in post-COVID-19 mucormycosis infections.^10^ However, the treating physician depends mainly on various clinical features and radioimaging details to adjust the dose and duration of antifungal drugs and the extent of surgery to reduce its extreme side effects. Few studies have evaluated the clinical and imaging criterion to develop a scoring system that can guide clinicians in the management strategy, but so far no consideration of histology findings has been analyzed.^11,12^ Thus, our objective was to evaluate the histology features and describe the associated clinical characteristics of invasive ROCM to provide a classification of severity of mucormycosis in post-COVID-19 patients. The study will be of importance for the optimal practice and treatment of mucormycosis.

## Materials and methods

The study was carried out in the Department of Pathology, R.D. Gardi Medical College, Central India, a 700-bedded rural referral teaching tertiary care hospital. All consecutive histopathology samples that were received during April–July 2021 from private facilities or from the Department of ENT of the hospital were included in the study. The demographic and clinical details of the patients such as signs, symptoms, site, imaging findings, laboratory parameters, such as complete blood count, blood sugar levels, C-reactive protein (CRP), serum ferritin, d-dimer, and KOH wet mount preparation, were noted in predesigned forms from the patient’s clinical sheets and the histopathology request form. The patient’s follow-up details as they survived or died were also noted. Patients who had antifungal treatment (oral or intravenous) within 72 h prior to surgical debridement were also included in the study.

**Figure 1.**
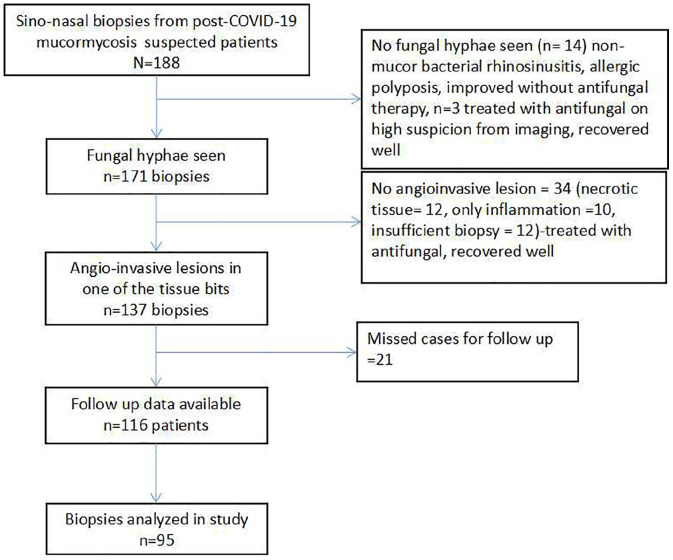
Overview of the cases enrolled in the study.

Microdebrider and forceps were used for good debridement of the tissue. Adequate representative samples (volume of tissue ranging from 2 to 16 g) were sent for histopathology in 10% neutral-buffered formalin. The tissue was examined for gross characteristics. At least 10 g of tissue was submitted in five blocks (2 g of tissue per cassette) for processing and if more tissue bits were available, an additional sample was processed when required (no invasion was seen on histology). The specimen for the KOH mount was sent separately in normal saline. Cases with non-invasive lesion on histology, insufficient tissue (less than 2 g) for grading and additional scoring, or specimen with only nasal crust removal were excluded from the final analysis. Patients whose survival follow-up data were missing were also excluded from the final analysis (Figure 1).

Five-micrometer-thick sections were stained with routine hematoxylin and eosin (H&E) stain and periodic acid Schiff (PAS) stain for histopathology evaluation and classification. The final diagnosis of the lesion was established on the basis of the histopathology characteristics. Patients with clinical features suggestive/suspicious of mucormycosis and showing the histopathology features of broad aseptate ribbon-like fungal hyphae with 90° branching were further evaluated. The slides were examined independently and blindly by three different pathologists. If there were differences in the classification between two pathologists, the histopathology findings were reviewed and discussed with the third pathologist to achieve consensus. The grading of severity classification of mucormycosis infection was assessed for the following four parameters (Table 2)^13^: (1) neutrophilic inflammatory cell infiltrates were assessed in tissue bits showing the presence of fungal hyphae in ×400 microscopic field; (2) degree of tissue necrosis was defined as the presence of non-viable tissue with fungal hyphae and was quantified as the percentage of the whole tissue under ×100 showing necrosis; (3) the fungal load was quantified as the number of fungal hyphae present in the ×400 field; and (4) number of blood vessels involved under ten ×400 microscopic fields. A consensus score of 1 to 3 or 1 to 2 was given on the assessment of microscopic examination of each parameter and the lesion was classified by adding up the scores. The lesion that scored in the range of 3–5 was graded I, 6–8 was graded as II, and grade III was given for a score of 9–11.

Ethical permission was sought from the institutional ethics committee (IEC-RDGMC-05/2021) and written informed consent was obtained from all patients or relatives before the study for participation and for any additional blood investigations which were performed for the sake of the study.

## Statistical analysis

We calculated the sample size for this rare lesion in specific post-COVID-19 patients based on assessing the proposed severity score and grade on histopathology of 20 debrided samples from ROCM. Angioinvasion was observed in 14 samples of which 41% cases were categorized to have grade III severity according to proposed scoring system. We calculated the minimal sample size of 93 cases for our study based on prevalence with 95% confidence interval and using the formula, n = z^2^ × P × (100 − P)/d^2^ (z = 1.96 at 95% confidence interval, P = 41% and d (absolute error) = 10%). Statistical analysis was performed using statistical software package Statistical Package for the Social Sciences (SPSS) version 21 (SPSS Inc., Chicago, IL, USA) and EPI-INFO version 6 (Centres of Disease Control and Prevention, Atlanta, Georgia, USA). Categorical variables were expressed as frequencies and percentages. The survival rate was calculated for all three grades. The Pearson chi-square test was used to compare the severity grade with their survival rates. The P value < 0.05 was considered statistically significant.

## Results

A total of 188 sino-nasal biopsies were identified from the patients with a suspected post-COVID-19 mucormycosis. Finally, biopsies showing mucormycosis with angioinvasive lesions with available follow-up data (N = 95) were analyzed in the study (Figure 1). The severity of histology grading was correlated with survival outcome.

A male predominance (74%) was observed with a male to female ratio of 2.8:1 (Table 1) at an age range of 26–75 years (mean age 46.8 ± 11 years). All patients had a history of COVID-19 disease in the last 2 months. Maximum cases (98%) presented within 20 days of COVID-19 treatment/recovery. All patients presented with one or other local or constitutional symptoms or signs. The most common complaint at the time of presentation was local facial pain (91.5%), swelling of the cheek (60%), and eye pain with periorbital swelling (29.5%). Other complaints at the time of presentation were epistaxis (in one patient with ipsilateral eye involvement), fever, numbness on the cheek skin, and moderate-to-severe toothache with or without loosening of the tooth. Most (70%) of the patients had symptoms for 5 days, while in 28% of the patients one of the symptoms was present for about 6–20 days. In 58% of the patients, right half of the face was involved, in 32% of the patients, left half of the face was involved, while 10% of the patients presented with bilateral disease.

**Table 1.** Demographic, clinical and investigation details of post-COVID-19 mucormycosis patients (N = 95).

	Number of cases	Percentage of cases
Age (years)		
21–40	24	25.2
41–60	48	50.5
61–80	23	24.2
Gender		
Male	70	73.6
Female	25	26.3
Clinical features		
Time from post-COVID-19 to onset of symptoms related to ROCM		
Within 20 days	93	98
20 days to 2 months	02	02
Symptoms related to ROCM		
Facial pain	87	91.5
Nasal pain with stuffiness	65	68.4
Cheek swelling	57	60
Fever	10	10.5
Pain in eye with orbital swelling	28	29.5
Pain in upper teeth	09	9.5
Pain in lower teeth with loosening	02	2.1
Severe headache	03	3.1
Side of involvement-right side	55	57.9
Left side	30	31.5
Bilateral	10	10.5
Uncontrolled diabetes mellitus	64	67.3
Laboratory parameters		
Neutrophilic leukocytosis^a^	67	70
Lymphopenia^b^	6	6
HbA1C^c^	51	53.6
Raised C-reactive protein^d^	85	89.4
Raised serum ferritin^e^	64	67.3
Raised d-dimer^f^	79	83.1
Fungal hyphae on KOH wet mount	59	62.1

ROCM: rhino-orbito- cerebral mucormycosis.

Reference range:

a Neutrophilic leukocytosis > 7000 neutrophils/µL.

b Lymphopenia < 800 cells/µL.

c Raised HbA1c > 7.

d Raised CRP > 6 mg/L.

e Raised serum ferritin > 137 ng/mL.

f Raised d-dimer > 500.

Diabetes was detected in 77% of the patients, among them 9% of the patients had newly detected diabetes. The mean HbA1C in diabetic patients was 7.4 ± 1.1. All patients had a history of steroid therapy during COVID-19 treatment and 67% had some or another type of assisted ventilation. Neutrophilic leukocytosis was observed in 70% of the patients with concomitant lymphopenia in 6% of the patients. Elevated blood d-dimer (523 ± 2.3 ng/mL), CRP (680 ± 34.4 mg/L), and ferritin levels (389 ± 74 ng/mL) were observed in 83.1%, 89.4%, and 67.3% patients, respectively. Wet KOH mount preparation was available for all the cases where 62.1% of the cases showed fungal hyphae with the Mucorales features and one case also showed microscopy features suggestive of candidiasis (Table 1).

Computed tomography (CT)/magnetic resonance imaging (MRI) of the sinus showed mucosal thickening (90% cases), bilateral sinus involvement (68% cases), bone destruction, black turbinate sign (16% cases), maxillary sinus involvement (98% cases), and ethmoid sinus (96% cases). The left side frontal sinus was almost twice as involved than the right side. Orbital involvement was present in 34% of the cases with muscle thickening and intracranial extension in three cases. Various clinical forms of the disease and the treatment strategy are shown in Supplemental Table 1.

**Figure 2.**
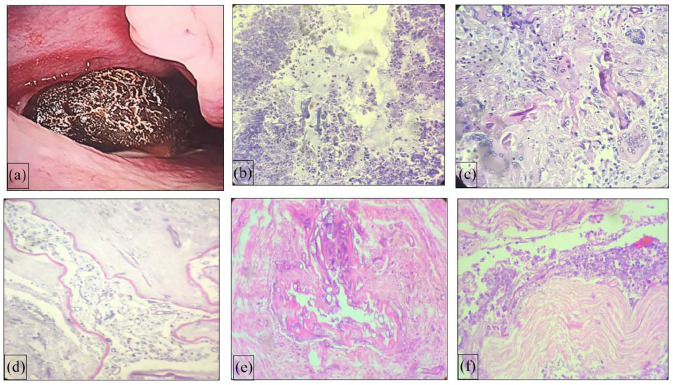
Photomicrograph showing broad aseptate hyphae of mucormycosis with (a) severe neutrophilic infiltrate, (b) giant cell reaction, (c) bone invasion, (d) angioinvasion, and (e) perineural invasion (H&E, ×400), (f) intraoperative nasal endoscopic finding of necrotic tissue in middle turbinate (zero-degree Hopkins nasal telescope).

The volume of tissue sent for histopathology examination ranged from 2 to 16 g, gray-white to black in color, and mainly soft to friable in consistency. Under the light microscope in H&E staining, all the cases showed characteristic broad, aseptate, ribbon-pattern, thin-walled, and irregular 90° branching fungal hyphae with inflammatory cell infiltrates in necrotic background. The underlying tissue reaction was predominantly suppurative with neutrophilic infiltrate, lymphocytes, plasma cells, and histiocytes. Only neutrophilic infiltrate was observed in 62% (n = 59) cases, neutrophil with lymphoplasmacytic inflammation was observed in 38% (n = 36) cases of which giant cell reaction was observed in 33.3% (n = 12) cases (Figure 2). The degree of neutrophilic infiltrate was assessed in tissue fragments having fungal hyphae (Figure 2). Neutrophil infiltrate was mild in 15.7% of the cases, moderate in 49.4%, and severe in 34.7% of the cases. None of the cases showed a complete absence of inflammation around the fungal hyphae. An ill-formed granuloma was seen in a non-diabetic patient with a fungal load of 2+ and grade 1 angioinvasion. However, no caseous necrosis was present in any of these cases. The Splendore–Hoeppli phenomenon was not detected in any of the cases. On PAS staining, similar broad hyphae were observed with weak positivity in 67% of the cases.

All cases showed tissue necrosis as basophilic amorphous material with fungal hyphae in one or all necrotic tissue bits. The degree of tissue necrosis was described as an assessed percentage of the area involved. Necrosis of ⩾50% tissue (grade 2) was observed in 58% of the cases. Necrotic bone pieces were seen in 94% of the cases. The fungal load was high (3+) in necrotic areas. The fungal load in areas of necrosis areas was 2+ in 40%, 3+ in 28.4%, 4+ in 21%, and 1+ in 10.5% cases.

The hyphae were characteristically seen around or in the wall or in the lumina of blood vessels (angioinvasion) and nerve bundles (Figure 2). Hyphae were mainly seen associated with veins although in some arteries were also involved. Angioinvasion of score 2 was observed in 68.4% of the cases, while 31.5% of the cases had angioinvasion of score 1. No vasculitis was seen in any section. Bone invasion was seen in six cases. Neural invasion was seen in three of the nine cases showing neural tissue on biopsy (3/9).

In cases that started with intravenous amphotericin within 72 h of surgical procedure, hyphae showed degenerative changes led to fragmentation, distortion, twisting, folding, and emptiness from within. A newly diagnosed diabetic patient with orbital involvement had a dimorphic fungal population of *Mucor* and *Aspergillus* on histopathology. Conidia of *Aspergillus* were also seen. Two other diabetic cases showed associated budding yeast forms with pseudo-hyphae suggesting mixed infection with candidiasis.

According to the histology criterion, the severity of the grade III infection was detected in 45.3% of the cases, grade II in 42.1% of the cases, and grade I in 12.6% of the cases (Table 2). Grade III severity with a maximum score of 11 was observed in four patients of whom three died from cerebral mucormycosis and one patient died from rhinosinusitis with orbital cellulitis; score of 10 (in four patients) and 8 (in two patients) was found in patients who had undergone orbital exenteration (n = 6). The patient who died of orbital exenteration with septicemia had a score of 10. In the statistical analysis, survival rate showed a significant correlation (p = 0.007) with the severity grade of histopathology.

**Table 2.** Histopathology score and grades of severity in post-COVID-19 mucormycosis patients (N = 95).

Histology parameter	Score	Number of cases n (%)
Fungal load^a^ (at ×400 microscopic field)		
<3 fields	1	10 (10.5)
3–5 fields	2	38 (40.0)
6–8 fields	3	27 (28.4)
>8 fields	4	20 (21.0)
Degree of angioinvasion (at ×400 microscopic field)		
<3 blood vessels in 10 fields	1	30 (31.5)
⩾3 blood vessels in 10 fields	2	65 (68.4)
Degree of tissue necrosis (at ×100 microscopic field)		
<50%	1	40 (42.1)
⩾50%	2	55 (57.8)
Neutrophilic infiltrate (at ×400 microscopic field)		
Mild	1	15 (15.7)
Moderate	2	47 (49.4)
Severe	3	33 (34.7)
Severity score		
Grade I	3–5	12 (12.6)
Grade II	6–8	40 (42.1)
Grade III	9–11	43 (45.3)

a Number of ×400 fields showing fungal hyphae.

## Discussion

During COVID-19 infection a tremendous increase in the incidence of ROCM and mortality has been observed in several parts of India compared to other countries affected with COVID-19.^6,4,7^ Ninety-eight percent of our cohort presented within 20 days of COVID-19 treatment as seen in a previous study.^14^ The higher prevalence of mucormycosis in India could be due to many possible reasons such as the presence of a high load of *Mucor* spores in the hospital and community due to the tropical and humid climate, the high prevalence of uncontrolled diabetes cases, the large number of latent diabetes cases, poor regular health check-up and monitoring blood sugar levels, newly developed diabetes due to COVID-19 virus attack on pancreatic cells, poor monitoring of the dose and duration of steroids used to treat COVID-19 cases, and poor maintenance of frequently used oxygen mask.^4,7,15,16^

We report the clinical and histopathology characteristics of mucormycosis in a series of post-COVID-19 patients. Uncontrolled diabetes, moderately severe pneumonia, mechanical ventilation, and steroid therapy were associated with the mucormycosis patients. Mucorales, being ubiquitous organisms, host characteristics are the most important factors in determining its pathogenesis. Most of the patients (77%) had underlying diabetes mellitus and received steroids.^15–18^ The presence of diabetes mellitus is an important predisposing factor for mucormycosis, as described in a meta-analysis among 851 patients with mucormycosis.^19^ Diabetic ketoacidosis causes fungal multiplication by increasing the concentration of free iron and decreasing the antifungal inhibitory factors in the serum.^20–22^ Neutrophilic leukocytosis and extreme lymphopenia (T cell more than B cell) in the presence of inhibitory cytokines and chemokines with a high and prolonged dose of steroid treatment administered to counteract the cytokine storm increased the incidence of mucormycosis in post-COVID-19 patients.^23^ Neutrophilic leukocytosis was relatively more common in our cases of ROCM compared to neutropenia, which is seen in pulmonary mucormycosis as previously seen.^15,16^ Steroids cause an increase in blood sugar levels in known diabetic patients and can switch pre-diabetics to the diabetic range. Some of the patients (9%) in our cohort also developed recent diabetes. In addition, the SARS-CoV-2 virus itself directly disturbs the β-cell integrity in pancreas and increases the risk of diabetes in patients of COVID-19.^24^ Altered natural killer cell activity, attenuated IFN-γ response, and hyperinflammatory state as evident with elevated CRP, d-dimer, and ferritin levels in our patients are seen primarily (80%) in diabetics. Increased levels of biomarkers are associated with a poor outcome.^25^ Increased use of tocilizumab, an immunosuppressive drug, in the COVID-19 treatment regime further predisposes patients to fungal infection.^26^

A proper diagnosis to distinguish mucormycosis from other bacterial and fungal infections is vital for early treatment and favorable outcomes. It has been shown that delayed initiation of therapy by only 6 days increases mortality by twofold.^27^ The success rates in the management of mucormycosis have ranged between 20% and 70% depending on the time lapse from presentation to diagnosis, the aggressive nature of the disease, and the immunocompromised state of the affected patients. A biopsy of sample from clinically affected sites and demonstration of hyphae by direct microscopy are crucial for diagnosis^13,28,29^ and to define and re-evaluate the treatment response. The presence of viable tissue along with necrotic tissue will confirm that a good debridement was performed. In the present case series, viable and necrotic tissue was seen in all cases (100%). Necrotic bone pieces were seen in 94% of the cases. Necrosis may be due to an infarction or inflammation. Infarction and hemorrhage are due to the angioinvasive nature of the hyphae that cause vessel wall destruction and luminal thrombosis.^24^ The inflammation response in mucormycosis can be seen in the form of giant cell reaction, as seen in 12.6% of our cases along with lymphocytes, plasma cells, histiocytic inflammation, neutrophilic infiltrate, or granulomas.^25^ Macrophages and neutrophilic oxidative bursts are the main host defense to kill proliferating hyphae. However, the use of steroids affects the phagocytic ability of macrophages and increases the risk of infection.^19,25^ The rare granulomatous response seen in post-COVID-19 mucormycosis could again be due to immune dysregulation as a result of steroid therapy. Occasionally, the hyphae are degenerated and broken on H&E stain. These hyphae are weakly positive on PAS staining. We believe that treatment is the cause of the presence of degenerated fungi and their presence should be mentioned in the patient’s report.

In our study, the presence of perineural fungal invasion was evaluated in all cases; however, only few cases (n = 9) showed the presence of neural tissue, out of which three cases showed the presence of fungal hyphae within it. Frater et al.^30^ had emphasized that perineural invasion by fungal hyphae should be ruled out before excluding the diagnosis of zygomycosis, as it is a preferential site of fungal growth.

Grading based on total scores for the extent of infection for each parameter, that is, degree and severity of inflammation, tissue necrosis, fungal load, and angioinvasion, showed significant correlation with the prognosis and survival rate (p = 0.007) of the patients. We not only assessed each parameter,^13^ but also graded the severity by the consensus added-up score so that all histology parameters can be taken into account for classification of a lesion. Thus, this approach can be a reliable aid to decide the prognosis in ROCM. The high density of fungal elements in necrotic tissue areas is negatively correlated with the survival rate. Thus, necrotic tissue should be well sampled and examined to detect fungal hyphae. Furthermore, angioinvasion and its severity were correlated with the outcome as in the previous study.^13,29^ Thus, our data suggest that the histology criterion should be used to design a scoring system and can be effectively incorporated into the few described clinical and radioimaging scoring systems to help in the decision-making of any extensive surgical intervention and debridement.^11,12^

The study has few limitations. The diagnosis of mucormycosis is recommended to be confirmed by tissue culture or by application of molecular or in situ identification techniques.^27^ However, these facilities are not available at all centers in resource-constrained settings. Also, contamination and very poor sensitivity are very common pitfalls with mucormycosis culture.^13,28^ Histology not only helps to give the diagnosis, but also defines the morphology of the tissue reaction, the morphology of the fungus, and the presence of tissue or blood vessel invasion.^27,28^ We nevertheless performed KOH mount as it is easy, simple, and even helps in immediate decision-making if performed per-operatively. In addition, considering the inherent nature of the study performed on the debrided specimen, subsites of debrided tissue are confounding factor at all times. However, scoring was not performed on samples that were inadequate or limited (less than 2 g) and showed only nasal crust. Maximum tissue was submitted for calculation of percentage of the tissue necrosis/fungal load/angioinvasion/inflammatory response as defined in the study. Furthermore, ours is single-center laboratory-based observational study with a limited number of samples from post-COVID-19 patients. However, our study provides information on a rare dreaded disease in specific post-COVID-19 patients for possible prognostic characteristics on histology.

## Conclusion

Our study emphasizes the need for a high index of suspicion for ROCM in post-COVID-19 patients. Grading based on tissue necrosis, degree of inflammation, fungal load, and angioinvasion helps to prognosticate the post-COVID-19 mucormycosis. Long-term multicentre follow-up study with larger samples will further help to validate the prognosis according to classification on histology.

## Supplemental Material

sj-doc-1-smo-10.1177_20503121221074785 – Supplemental material for Clinical and histology features as predictor of severity of mucormycosis in post-COVID-19 patients: An experience from a rural tertiary setting in Central India Click here for additional data file. Supplemental material, sj-doc-1-smo-10.1177_20503121221074785 for Clinical and histology features as predictor of severity of mucormycosis in post-COVID-19 patients: An experience from a rural tertiary setting in Central India by Kavita Jain, Akshay Surana, Tej Singh Choudhary, Sudhakar Vaidya, Shirish Nandedkar and Manju Purohit in SAGE Open Medicine
