# Supplementary material for: Clinical and histology features as predictor of severity of mucormycosis in post-COVID-19 patients: An experience from a rural tertiary setting in Central India
Source: SAGE Open Med. 2022 Feb 3;10:20503121221074785. doi: 10.1177/20503121221074785 (PMC8819781; doi:10.1177/20503121221074785)
Supplement: sj-doc-1-smo-10.1177_20503121221074785 – Supplemental material for Clinical and histology features as predictor of severity of mucormycosis in post-COVID-19 patients: An experience from a rural tertiary setting in Central India [file sj-doc-1-smo-10.1177_20503121221074785.doc]

**Suppl table 1 Clinical forms and management** details of post-COVID-19 mucormycosis patients (N=95)

| Clinical management of patients | Rhinosinusitis  n=53 | Rhinosinusitis with orbital cellulitis  n=28 | | Maxillary mucormycosis limited to upper alveolus  n =06 | Maxillary mucormycosis  n =03 | Mandibular mucorycosis  n =02 | Rhinosinusitis with intracerebral complication  n=03 |
| --- | --- | --- | --- | --- | --- | --- | --- |
| Time lapse between post-COVID 19 treatment and diagnosis of mucormycosis (days) | 6 to 15 | 4 to 7 | | 10 to 15 | 10 to 15 | 30 to 45 | 6 to 10 |
| Surgical procedure performed | *FESS with debridement | FESS with orbital decompression | | Partial maxillectomy | Total maxillectomy | Mandibular curettage | FESS with debridement |
| Antifungal treatment | Amphotericin B followed by Posaconazole | | | | | | |
| Doses | Lipohilic Amphotericin B: 1 mg/kg body weight or Liposomal Amphotericin B: 4- 5mg/ kg body weight | | | | | | Lipohilic Amphotericin B: 1.5 mg/kg body weight or Liposomal Amphotericin B: 6 mg/ kg body weight |
| Duration of treatment (days) | 20 to 25 | 20 to 25 | | 30 | 30 | 45 | 10 to 15 |
| Follow up | ^#^DNE every 7th day clear crust, review histopathology | | | Endoscopy/oral examination every 7th day clear crust, review histopathology | | | Repeated cerebral imaging, endoscopy/ oral examination every day, on 7th day clear crust and histopathology |
| Outcome of the disease | One patient died of septicemia | | Orbital exenteration in six patients, one patient died of septicemia | All recovered | All recovered | All recovered | All died |

*FESS-Functional endoscopic sinus surgery; ^#^DNE-diagnostic nasal endoscopy
